# The complexity underlying treatment rankings: how to use them and what to look at

**DOI:** 10.1136/bmjebm-2021-111904

**Published:** 2022-05-02

**Authors:** Virginia Chiocchia, Ian R. White, Georgia Salanti

**Affiliations:** 1 Institute of Social and Preventive Medicine, University of Bern, Bern, Switzerland; 2 Graduate School of Health Sciences, University of Bern, Bern, Switzerland; 3 Medical Research Council Clinical Trials Unit, University College London, London, UK

In clinical fields where several competing treatments are available, network meta-analysis (NMA) has become an established tool to inform evidence-based decisions.^1 2^ To determine which treatment is the most preferable, decision-makers must account for both the quantity and the quality of the available evidence by considering both efficacy and safety outcomes as well as assessing the confidence in the obtained results.^3^ It is, however, increasingly common to include in the NMA output a ranking of the competing interventions for a specific outcome of interest.^4^ This article focuses on this type of rankings.

A hierarchy of treatments (or ranking) is obtained by ordering a specific ranking metric. A ranking metric is a statistic measuring the performance of an intervention and is calculated from the estimated relative treatment effects and their uncertainty in NMA.^5^ A commonly used ranking metric is the point estimate of the relative treatment effects against a natural common comparator such as placebo. The rankings are unaffected by choice of comparator, so any comparator may be chosen.^6^ Other commonly used metrics are the probability of producing the best outcome value, *p_BV_* (sometimes called probability of being the best), and the surface under the cumulative ranking curve (SUCRA) or their frequentist equivalent, the P-score.^7^ Treatment hierarchies are a simple and straightforward way to display the relative performance of an intervention and aid the decision-making process, so nowadays most publications and reports present rankings.^4^ Furthermore, new ranking metrics are being developed to obtain treatment hierarchies that account for important clinical and methodological aspects, such as multiple outcomes (benefits and risks), clinically important differences and the quality of the evidence.

Ranking metrics have been criticised in the literature for their lack of reliability, quoting, among other issues, limited interpretability and ‘instability’.^8–11^ This criticism was based on the disagreement between hierarchies obtained by the different ranking metrics. Consider for example the different treatment hierarchies in figure 1 obtained by different ranking metrics for a network of nine antihypertensives for primary prevention of cardiovascular disease^12 13^ (network graph shown in figure 2). The treatment hierarchy based on *p_BV_* disagrees markedly with the other hierarchies, based on relative treatment effects and *SUCRA*, particularly with respect to the top treatment. Conventional therapy, an ill-defined treatment which was evaluated in only one trial, is in the first rank in the hierarchy based on *p_BV_* but only in the third/fourth and sixth rank in the hierarchies according to the relative treatment effects and *SUCRA*, respectively.

Although such examples can occur, a recent empirical study showed that they are rather rare and that in general there is a high level of agreement between the hierarchies produced by the most common ranking metrics.^13^ Agreement becomes less when, as in the network of anti-hypertensives, there are large differences in the precision between the treatment effect estimates. These differences in precision could be produced by different data features, such as sparse or poorly connected networks, heterogeneity and inconsistency.^14^ Disagreements mostly relate to hierarchies based on *p_BV_*. Salanti *et al* also showed with theoretical examples how the uncertainty in the estimation of the relative treatment effects may affect the order of treatments in a ranking. In particular, they observed how rankings based on *p_BV_* are more sensitive to differences in precision across treatment effect estimates than those based on SUCRA. When competing treatments have similar point estimates, *p_BV_* tends to rank first the treatment with the most imprecise effect (largest confidence or credible interval); a high *p_BV_*, therefore, tends to accompany a high probability of producing the worst value. This observation is confirmed by the empirical results in Chiocchia *et al*^13^ and can easily be seen in the antihypertensive treatments example where the conventional therapy drops several ranks in the hierarchy based on SUCRA (figure 1). As displayed by the relative treatment effects of overall mortality for each treatment versus placebo in the forest plot in figure 3, the point estimates are all quite similar but the risk ratio of conventional therapy vs placebo is the only one with a large degree of uncertainty. This very imprecise effect and the large differences in the precision of the treatment effect estimates lead to the conventional therapy being the top treatment according to the *p_BV_* ranking and to the disagreement between the latter and the other two rankings.

It is important to point out that all ranking metrics are statistics calculated from the data and none of them provides a ‘gold standard’ against which each other ranking metric should be evaluated. Consequently, the criticism that some of the resulting treatment hierarchies are unreliable and unstable because they do not agree with other hierarchies is misplaced. But then, which hierarchy should one report and use to make decisions? The appropriate treatment hierarchy to use is the one resulting from the metric that answers the ‘treatment hierarchy question’ that the systematic review is posing.^14^ For example, if we are interested in ‘which treatment is the most likely to produce the largest positive change in the outcome’ (eg, relative drop in blood pressure or increase in quality of life) then *p_BV_* will lead to the relevant treatment hierarchy. However, we think this is not the relevant treatment hierarchy question for patients. If we want to know ‘which treatment is likely to outperform most competitors?’ then we should employ *SUCRA* rankings. Salanti *et al* report some examples of treatment hierarchy questions for rankings based on the most popular ranking metrics.^14^ These questions and the way they are phrased are, however, not set in stone as they are suggestions based on the most common approaches and decision-making problems. Further research is needed in the field to understand what most patients and clinicians expect when they ask about the ‘best treatment’.

Even with a careful choice of ranking metric, the treatment at the top of the resulting treatment hierarchy may not necessarily reflect the ‘best clinical choice’. Rankings cannot be used to understand whether differences between the interventions are clinically important or not. Rankings on their own have little meaning if not presented side-by-side with measures that quantify the differences in clinical outcomes, such as mean differences or risk ratios, often presented in league tables.^15^ Several choices need to be made in the full decision-making context: what outcomes are important and how do we trade-off between them? Do the observed differences reflect clinically important differences? What aspect do patients and/or clinicians value the most? How confident are we in the NMA results? These are only some of the aspects that must be considered in the complex decision-making process. New ranking approaches have been developed to address these questions. Multicriteria decision analysis is a comprehensive methodology that incorporates preference information with a benefit-risk assessment identified by explicit tradeoffs across multiple outcomes.^16 17^ The P-score^7^ was extended to account for clinically important relative differences on more than one outcome^18^ while Spie charts can be used to visualise compartive effectiveness and safety on multiple outcomes of equal or different importance to a decision-maker.^19^ The Probability of Selecting a Treatment to Recommend incorporates important information such as the confidence in the evidence or clinical priors in the ranking algorithm.^20^ A first approach to evaluate the confidence in rankings from NMA was described by Salanti *et al* but it has not yet been implemented into a proper framework like CINeMA.^3 21^ The aim to create evidence-based guidelines also inspired the threshold analysis approach, which is not a new ranking method per se, but it informs on the robustness of treatment recommendations by quantifying how much the evidence could change before the ranking of the treatments changes.^22^ In view of these new methods, NMA has the potential to provide answers to more comprehensive and complex treatment hierarchy questions and aid the decision-making process more efficiently.

If obtaining a treatment hierarchy is one of the aims of the synthesis, we recommend reviewers to specify the treatment hierarchy question a priori in the protocol, together with the appropriate ranking metric to answer that treatment hierarchy question. This is the first step to avoid misinterpreting the findings of the chosen ranking. The presented treatment hierarchy must be interpreted together with the relative treatment effects, with particular attention to the uncertainty in the estimations, as well as the quality of the synthesised evidence. More work focusing on the development of a comprehensive framework for evaluating the confidence in the rankings of treatments is needed.

## Figures and Tables

**Figure 1:**
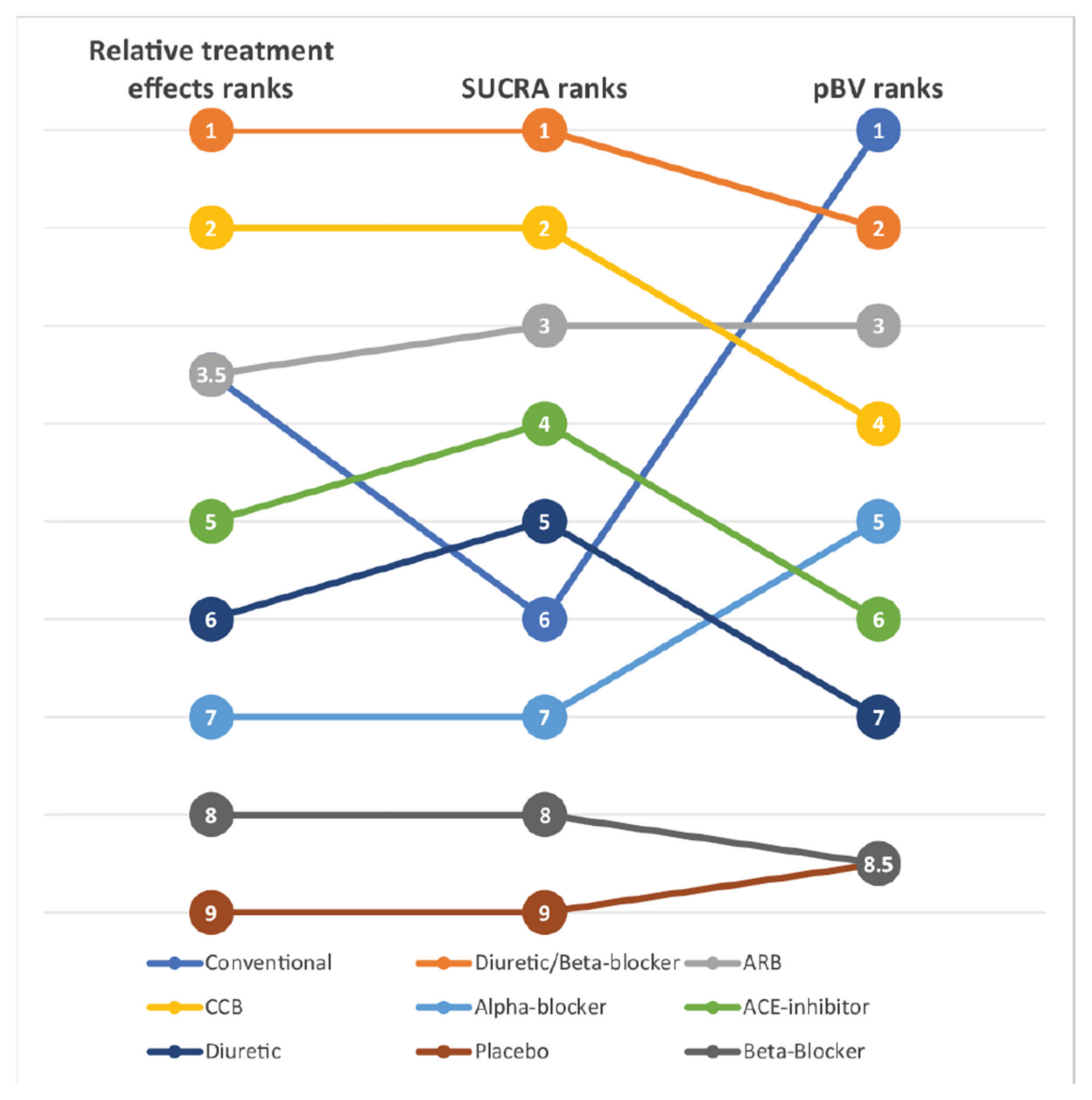
Example of treatment hierarchies from different ranking metrics for a network of nine antihypertensive treatment for primary prevention of cardiovascular disease. ARB, angiotensin receptor blockers; CCB, calcium channel blockers; *p_BV_*, probability of producing the best value; *SUCRA*, surface under the cumulative ranking curve (calculated in frequentist setting).

**Figure 2:**
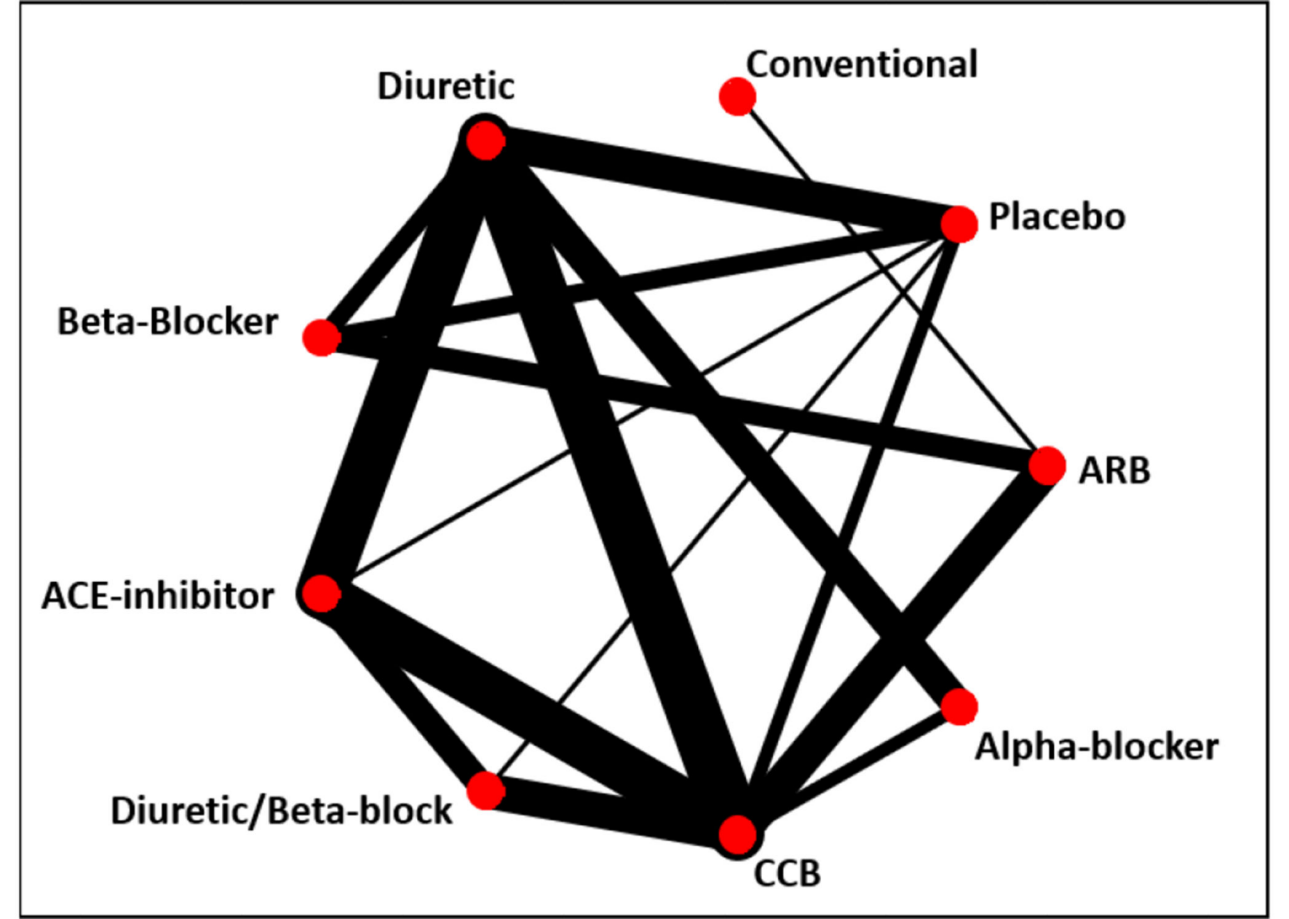
Graph of network of nine antihypertensive treatments for primary prevention of cardiovascular disease. line width is proportional to inverse SE of estimates from random effects model comparing two treatments. ARB, angiotensin receptor blockers; CCB, calcium channel blockers.

**Figure 3:**
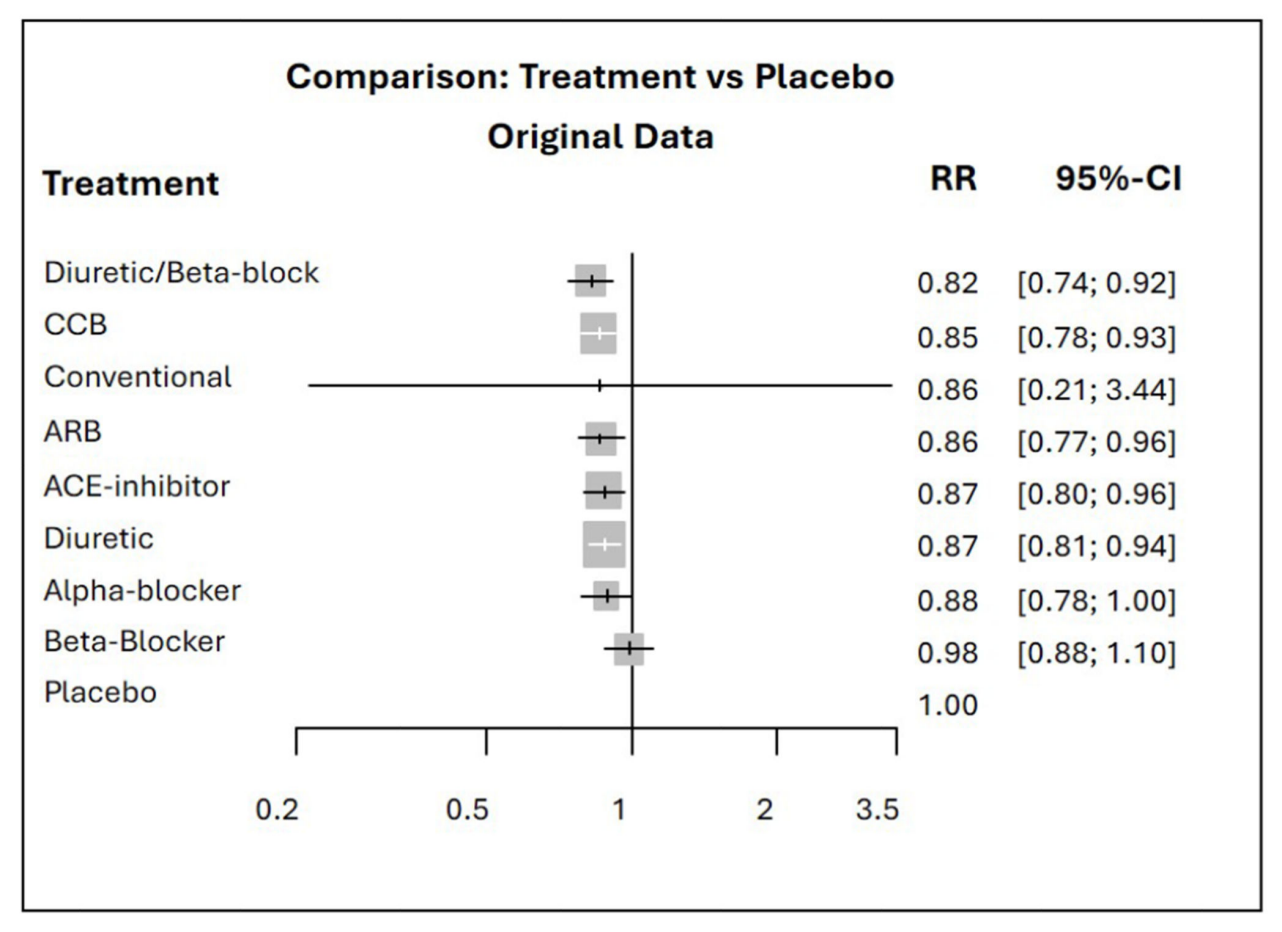
Forest plots of relative treatment effects of overall mortality for each treatment vs placebo. ARB, angiotensin receptor blockers; CCB, calcium channel blockers; RR, risk ratio.

## Highlights/key points

⇒ Treatment hierarchies obtained by SUCRA, p_BV_, mean ranks and mean relative effects might differ when there are large differences in the amount of data for each treatment
⇒ Different hierarchies do not imply that one is wrong or better than the others, because the methods used to rank treatments address different ‘treatment hierarchy questions’ based on how the ‘preferable treatment’ is defined
⇒ The treatment at the top of the ranking may not reflect the ‘best clinical choice’: rankings must be considered together with relative treatment effects and quality of the evidence
⇒ Researchers should specify in the protocol whether among the aims of the synthesis is to obtain a treatment hierarchy and, if yes, which is the ‘treatment hierarchy question’ they aim to answer
